# Histone Monoubiquitination in Chromatin Remodelling: Focus on the Histone H2B Interactome and Cancer

**DOI:** 10.3390/cancers12113462

**Published:** 2020-11-20

**Authors:** Deborah J. Marsh, Yue Ma, Kristie-Ann Dickson

**Affiliations:** 1 Translational Oncology Group, Faculty of Science, School of Life Sciences, University of Technology Sydney, Ultimo, NSW 2007, Australia; Yue.Ma-7@student.uts.edu.au (Y.M.); Kristie-Ann.Dickson@uts.edu.au (K.-A.D.); 2 Kolling Institute, Faculty of Medicine and Health, Northern Clinical School, University of Sydney, Camperdown, NSW 2006, Australia

**Keywords:** histone monoubiquitination, E3 ubiquitin ligases, RNF20, p53, BRCA1, CDC73, SWI/SNF, transcriptional elongation, DNA damage

## Abstract

**Simple Summary:**

Post-translational modifications (PTM) of histone tails represent epigenomic regulation of the chromatin landscape, influencing gene expression and the response to DNA damage. This review focusses on cancer-associated roles of ubiquitin as a histone PTM, specifically in conjunction with an E3 ubiquitin ligase cascade that results in the addition of a single ubiquitin (monoubiquitination) to histone H2B at lysine 120 (H2Bub1). H2Bub1 has roles in chromatin accessibility important for transcriptional elongation, the DNA damage response, cellular proliferation and developmental transitions, including in stem cell plasticity. It has been implicated in inflammation and tumour progression, with examples of its loss associated with a worse prognosis for patients with some cancers. Many factors involved in the H2Bub1 interactome are well known cancer-associated proteins, including p53, BRCA1 and components of the SWI/SNF remodelling complex. Increased knowledge of H2Bub1 and its interactome offers new opportunities for therapeutic targeting of malignancy.

**Abstract:**

Chromatin remodelling is a major mechanism by which cells control fundamental processes including gene expression, the DNA damage response (DDR) and ensuring the genomic plasticity required by stem cells to enable differentiation. The post-translational modification of histone H2B resulting in addition of a single ubiquitin, in humans at lysine 120 (K120; H2Bub1) and in yeast at K123, has key roles in transcriptional elongation associated with the RNA polymerase II-associated factor 1 complex (PAF1C) and in the DDR. H2Bub1 itself has been described as having tumour suppressive roles and a number of cancer-related proteins and/or complexes are recognised as part of the H2Bub1 interactome. These include the RING finger E3 ubiquitin ligases RNF20, RNF40 and BRCA1, the guardian of the genome p53, the PAF1C member CDC73, subunits of the switch/sucrose non-fermenting (SWI/SNF) chromatin remodelling complex and histone methyltransferase complexes DOT1L and COMPASS, as well as multiple deubiquitinases including USP22 and USP44. While globally depleted in many primary human malignancies, including breast, lung and colorectal cancer, H2Bub1 is selectively enriched at the coding region of certain highly expressed genes, including at p53 target genes in response to DNA damage, functioning to exercise transcriptional control of these loci. This review draws together extensive literature to cement a significant role for H2Bub1 in a range of human malignancies and discusses the interplay between key cancer-related proteins and H2Bub1-associated chromatin remodelling.

## 1. Introduction

Post-translational modifications (PTMs) of core histone proteins H2A, H2B, H3 and H4 constituting the nucleosome have driving roles in modulating the chromatin landscape in order to regulate fundamental processes such as transcription and the DNA damage response (DDR). Examples of histone PTMs include methylation, acetylation, phosphorylation, SUMOylation, proline isomerisation and ubiquitination [1,2]. The addition of a single 76 amino acid (8.5 kDa) ubiquitin, monoubiquitination, is one of the bulkier histone modifications [2,3]. A number of histone lysines (K) are monoubiquitinated in the mammalian genome, including K13, K15, K119, K127 and K129 of histone H2A; K34, K120 and K125 of histone H2B; and K31 on histone H4 (reviewed in [4]). The two most well-known mammalian histone monoubiquitination events occur on K119 of histone H2A (H2AK119ub1) linked to the Polycomb Repressor Complex 1 (PRC1) [5] and K120 of histone H2B (H2Bub1), the latter being the subject of this review. Interestingly, these events have opposing functions, with H2AK119ub1 associated with gene silencing and H2Bub1 frequently associated with active transcription [4,5,6].

**Figure 1:**
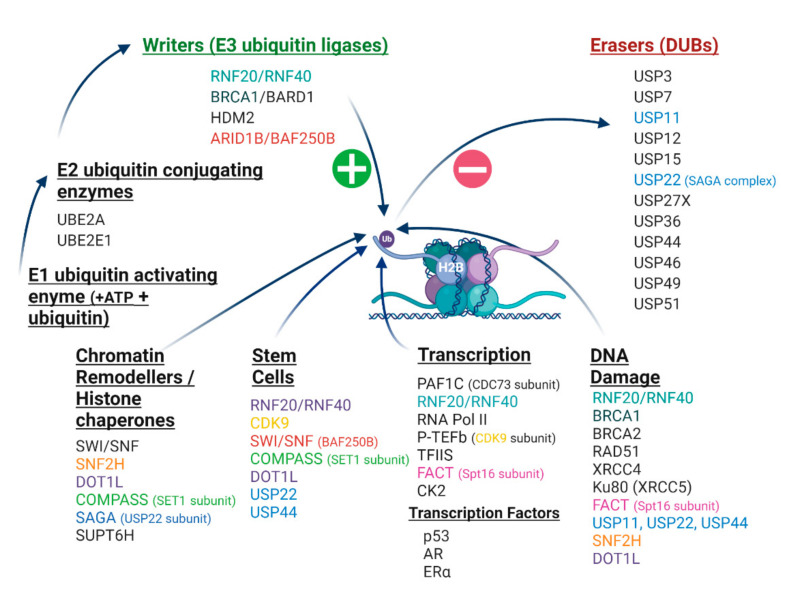
The H2Bub1 interactome. H2Bub1 can be written (green plus symbol) by multiple writer enzymes (E3 ubiquitin ligases) following an enzymatic ubiquitin cascade commencing with an E1 ubiquitin activating enzyme followed by an E2 ubiquitin conjugating enzyme. It can be removed (red minus symbol) by numerous erasing deubiquitinases (DUBs) from the Ubiquitin-Specific Protease (USP) sub-family. H2Bub1 interacts with a number of chromatin remodelling complexes and histone chaperones. Numerous components of the H2Bub1 interactome function in more than one cellular process, such as cyclin-dependent kinase 9 (CDK9) in stem cell plasticity and transcription, identified in this schematic by matching colours. The H2Bub1 interactome continues to be elucidated and is growing as new discoveries are made. Androgen receptor (AR); AT-Rich Interaction Domain 1B (ARID1B); BRG1/BRM-associated factor (BAF250B); BRCA1-Associated RING Domain 1 (BARD1); Breast Cancer Type 1 (BRCA1); Breast Cancer Type 2 (BRCA2); cyclin-dependent kinase 9 (CDK9); estrogen receptor alpha (ERα); complex of proteins associated with SET1 (COMPASS); disrupter of telomere silencing 1-like (DOT1L); facilitates chromatin transcription (FACT); Human Double Minute 2 (HDM2); X-ray Repair Cross Complementing 5 (Ku80, also known as XRCC5); RNA polymerase II-associated factor 1 complex (PAF1C); Positive Transcription Elongation Factor-b (P-TEFb); tumour protein 53 (p53); RNA polymerase II (RNA Pol II); RAD51 Recombinase (RAD51); RING Finger Protein 20 (RNF20); RING Finger Protein 40 (RNF40); Spt-Ada-Gcn5-acetyltransferase (SAGA); Su(var) 3–9 (SET1, suppressor of position effect variegation), enhancer of zeste (E(z)), and trithorax (Trx); SNF2 homologue, (SNF2H, also known as SMARCA5); Suppressor of Ty Homologue-6 (SUPT6H); Switch/sucrose non-fermenting (SWI/SNF); transcription elongation factor II S (TFIIS); ubiquitin conjugating enzyme E2 A (UBE2A); ubiquitin-conjugating enzyme E2 E (UBE2E1); Ubiquitin-Specific Protease (USP); X-ray Repair Cross Complementing 4 (XRCC4). Created with BioRender.com.

A cascade of enzymatic events is responsible for monoubiquitination, involving an activating ATP-dependent ubiquitin enzyme E1 of which humans have a few, a ubiquitin conjugating enzyme (E2) of which humans have around 40, and a ubiquitin protein ligase (E3) of which humans have as many as 600–1000 [4,7,8]. The most prevalent of the E3 ubiquitin ligases are RING (really interesting new gene) finger domain ligases of which the E3 complex RNF20/RNF40 is the main writer enzyme complex responsible for catalysing H2Bub1 [1,9]. The H2Bub1 chromatin mark is erased by deubiquitinases (DUBs) from the ubiquitin-specific protease (USP) sub-family of DUBs, including USP44 and USP22 (reviewed in [1,10,11,12]) (Figure 1).

The enzymatic cascade that writes H2Bub1 is invoked to enable association of the human RNA polymerase II-associated factor 1 (PAF1) transcriptional complex with RNA polymerase II (RNA pol II) to facilitate transcriptional elongation [13]. It is also required to facilitate the accumulation of H2Bub1 at sites of double strand breaks (DSBs) where it functions as a scaffold or docking platform for the recruitment of DNA repair factors [14]. H2Bub1 has also been linked to DNA replication in yeast [15] and in both yeast and human cells, genomic stability through the maintenance of centromeric chromatin [16]. While H2Bub1 itself has been described as having tumour suppressive properties, a number of tumour suppressor proteins are also associated with H2Bub1-related processes including RNF20, CDC73, BRCA1, p53 and members of the switch/sucrose non-fermenting (SWI/SNF) chromatin remodelling complexes (also known as the BAF complexes) [17,18,19,20].

Global levels of H2Bub1 and/or its E3 ligase complex members RNF20 and RNF40, have been investigated in numerous tumours using methods including immunohistochemistry and for RNF40 and/or RNF20, assessment of transcript levels and promoter hypermethylation. Dependent upon tumour type, depleted global H2Bub1 levels have been shown to correlate with inflammatory processes that may function as precursor events to tumorigenesis, in tumour progression and in some tumour types have been correlated with a worse prognosis [21,22]. 

On the background of global loss of H2Bub1 in cancer cells, H2Bub1 is enriched at the coding regions of highly expressed genes in response to DNA damage, including p53 gene targets and genes involved in resistance to therapeutic drugs [23,24]. H2Bub1 therefore has a role in determining the fate of cancer cells that may be amenable to therapeutic intervention. Further evidence for a key role in determining cell fate is the role of H2Bub1 in the differentiation of stem cells, whether it be through engaging with other histone PTMs via histone cross-talk, or the enzymes involved in writing and erasing H2Bub1 [10,12]. H2Bub1 and its associated factors have also been linked to regulation of key development pathways in plants, including seed dormancy/germination and flowering time, as well as resistance to pathogen invasion [25,26,27,28]. It is clear that H2Bub1 has a significant role across species to regulate important functions through the modulation of gene expression.

In this review we discuss the role of H2Bub1 in fundamental cellular processes including gene transcription, the DDR and stem cell differentiation. We bring together the current literature on the involvement of H2Bub1 across multiple different tumour types, including links recently established between H2Bub1 and inflammation. Lastly, we investigate the current state-of-play between H2Bub1 and members of its interactome complicit in the establishment and/or progression of malignancy. 

## 2. H2Bub1 and Transcriptional Elongation

Monoubiquitination isn’t the only histone PTM that occurs at K120 of histone H2B. Acetylation at this site (H2BK120ac) written by the lysine acetyl transferase KAT3 is thought to act as a mark of chromatin poised to enter the active state, highlighting the temporal acetylation/ubiquitination switch working to achieve transcription [29]. H2Bub1 physically disrupts chromatin compaction, creating a more open conformation accessible to transcription factors and other proteins and/or protein complexes involved in activities such as DNA repair [30]. This is more than purely physical disruption, as replacement of ubiquitin with the even bulkier molecule small ubiquitin-like modifier (SUMO), does not result in the same functional effects [31]. K120 of histone H2B is physically located at the interface of adjacent nucleosomes, making it possible that H2Bub1 has an impact on nucleosome stacking that may affect nucleosome stability [30]. Chandrasekharan and colleagues demonstrated that nucleosome stability increases in the presence of H2Bub1 during transcription and that this negatively impacts upon cell growth [31], with a later study suggesting that this may be a modest effect [32].

H2Bub1 is enriched at the coding regions of highly expressed genes [24]. In response to stimuli such as a hormone and/or a developmental signal or DNA damaging agent, cyclin-dependent kinase 9 (CDK9), part of the Positive Transcription Elongation Factor-b (P-TEFb) [33] complex, phosphorylates both the H2Bub1 E2 enzyme UBE2A and Ser2 in the carboxy-terminal domain (CTD) of RNA Pol II [12,34]. This creates a binding domain for WAC (WW Domain-Containing Adapter Protein with Coiled-Coil) that directly links the main H2Bub1 E3 ligase complex RNF20/RNF40 to RNA pol II [12,35]. In this co-transcriptional role with H2Bub1, CDK9 has been shown in yeast to be responsible for releasing RNA pol II from promoter-proximal pausing, making it an important regulator of gene expression [1,36,37]. Notably, RNA pol II promoter-proximal pausing has been implicated in the suppression of transcription of antisense genes [38].

**Figure 2:**
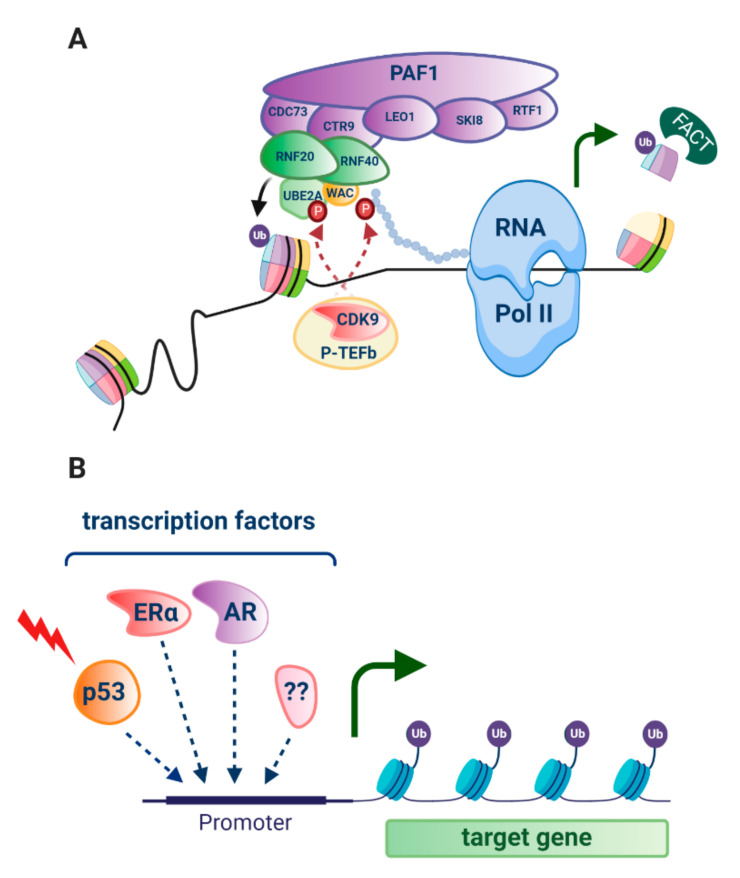
H2Bub1 in transcriptional elongation. (**A**) Following stimuli such as DNA damage, hormones or developmental signals, cyclin-dependent kinase 9 (CDK9) that is part of the Positive Transcription Elongation Factor-b (P-TEFb) complex phosphorylates Ser2 in the carboxy-terminal domain of RNA polymerase II (RNA pol II) depicted by a chain of circles, as well as the E2 ubiquitin conjugating enzyme ubiquitin conjugating enzyme E2 A (UBE2A). This dual phosphorylation shown by red circles, establishes a binding domain for the WW domain-containing adaptor with coiled-coil (WAC) and the E3 ubiquitin ligase complex RNF20/RNF40 that monoubiquitinates histone H2B at lysine 120 (H2Bub1, ubiquitin shown as a purple circle). RNF20/RNF40 physically interacts with the PAF1 complex consisting of CDC73, CTR9, LEO1, SKI8, RTF1 and PAF1 that associates with RNA pol II to establish transcriptional elongation. The chromatin remodelling factor FACT is recruited by H2Bub1, and removes a H2B-H2A dimer from the core nucleosome that takes away the physical block to RNA pol II, allowing it to move through the nucleosome, facilitating gene expression (green arrow). (**B**) A number of transcription factors have been associated with enriched H2Bub1 at target genes, including p53 (red lightning bolt depicts DNA damage that activates this tumour suppressor), the estrogen receptor (ERα) and androgen receptor (AR). ?? indicates as yet unknown transcription factors. Created with BioRender.com.

Both RNF20 and RNF40 have been shown to physically interact with subunits of the human PAF1C (including the PAF1 subunit itself, CDC73 and CTR9) that associates with RNA Pol II, setting the stage for interaction of all these factors that enable transcriptional elongation to proceed [39] (reviewed in [1,10,12]) (Figure 1, Figure 2A). Enrichment of H2Bub1 occupancy at highly expressed genes has been shown to correlate with the recruitment of Pol II, prior to the increase of mRNA expression and to decrease once RNA Pol II has dissociated [24]. Examples of genes with enriched H2Bub1 include those that are transcriptionally driven by hormone receptors, specifically estrogen receptor alpha (ERα) [22,40,41] important in some breast and ovarian cancers, and the androgen receptor (AR), important in prostate cancer [42], as well as p53 target genes that function in the cellular response to DNA damage [10,24] (Figure 2B). 

To enable transcriptional elongation, H2Bub1 recruits the histone chaperone that facilitates chromatin transcription (FACT) that works to remove the H2A-H2B dimer from the nucleosome, therefore removing the physical block to RNA pol II (Figure 2A) [43]. A FACT subunit, Spt16, functions with H2Bub1 to then enable nucleosome reassembly following RNA pol II transcriptional elongation [44]. These apparently opposing functions of H2Bub1 highlight its key roles in the kinetics of nucleosome assembly and disassembly required during and post transcription. 

H2Bub1 is also central in trans-histone crosstalk, recruiting histone methyltransferase complexes that further direct chromatin configuration and gene expression [45,46,47,48]. The catalytic activity of the disrupter of telomere silencing 1-like (DOT1L) histone methyltransferase that facilitates methylation of histone H3 at K79 (H3K79me) at the proximal region of actively transcribed genes is stimulated by H2Bub1 [49,50,51,52,53,54]. Loss of H3K79me2 has been correlated with genomic instability [55]. Another active chromatin mark is histone H3K4 methylation that is catalysed by SET1, the methyltransferase subunit of the complex of proteins associated with SET1 (COMPASS) after first being recruited by H2Bub1 [39,56,57]. Furthermore, Basnet and colleagues report an association between the phosphorylation of tyrosine 57 on histone H2A by casein kinase 2 (CK2) and H2Bub1 in yeast and mammalian cells. Loss of this histone H2A phosphorylation event, either by mutation at the histone H2A tyrosine site or inhibition of CK2, leads to loss of H2Bub1, H3K4me3 and H3K79me3, impacting on transcriptional elongation and likely involving the Spt-AdaGcn5 acetyltransferase (SAGA) chromatin modifying complex [58]. This is another example of how H2Bub1 functions in histone crosstalk. These patterns of trans-histone cross-talk cement the importance of H2Bub1 as a central regulator of transcription (Figure 1).

While being a clear enabler of transcriptional elongation, in certain circumstances H2Bub1 can also impede it. At least in yeast, there would appear to be a positional effect determined by gene location given that H2Bub1 at promoters of genes that aren’t expressed appear to inhibit the formation of transcriptional complexes [59]. The H2Bub1 writer enzyme RNF20 has been demonstrated to obstruct binding of the transcription elongation factor transcription elongation factor II S (TFIIS) to the PAF1C, so blocking the ability to relieve stalled RNA pol II on chromatin [60]. In this way, RNF20 works to selectively inhibit the transcription of pro-oncogenic genes located in condensed chromatin, supportive of a tumour suppressor function for this E3 ligase. H2Bub1 occupancy is, therefore, complex and likely tissue, genomic position and contextually specific, as not all genes require H2Bub1 enrichment for their expression [61]. One study combining RNA sequencing and Assay for Transposase Accessible Chromatin (ATAC-seq) in fallopian tube epithelial cell lines suggested an association between loss of H2Bub1 and a more open chromatin configuration [62]. In summary, H2Bub1 is a master regulator of transcription, controlling gene expression in response to acute stimuli and developmental signals in a context specific manner and occupying a central role in histone cross-talk directing gene expression.

## 3. H2Bub1 and the DNA Damage Response (DDR)

**Figure 3:**
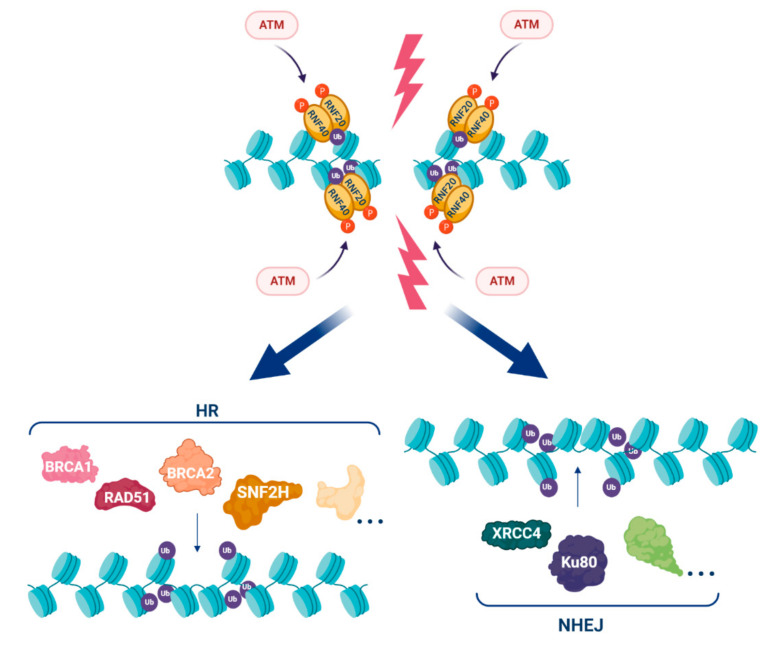
H2Bub1 in the DNA damage response. In the presence of double strand breaks (DSBs), ATM (ataxia telangiectasia mutated) phosphorylates (red circles) RNF20 and RNF40 that localise to DSBs where they function as an E3 ligase complex to write H2Bub1 (purple circles). H2Bub1 acts as a platform to attract proteins that work in both homologous recombination (HRR: BRCA1, BRCA2, RAD51, SNF2H and additional proteins) and non-homologous end joining (NHEJ: XRCC4, Ku80 (XRCC5) and additional proteins) to enable DNA repair. Created with BioRender.com.

H2Bub1 accumulates after DNA damage at sites of DSBs as part of the cellular DDR [14,63,64]. Upon DNA damage, the ATM (ataxia telangiectasia mutated) kinase phosphorylates RNF20 and RNF40, facilitating the recruitment of this E3 ligase complex to DSBs where it acts to catalyse H2Bub1 [14]. DNA damage-associated H2Bub1 then acts as a platform to recruit the chromatin remodelling factor SNF2H and other proteins required for homologous recombination repair (HRR) including BRCA1, BRCA2 and RAD51, as well as non-homologous end joining, including XRCC4 and Ku80 (Figure 1 and Figure 3) [14,63,65].

Numerous factors that have a role in H2Bub1-related transcription also have a role in H2Bub1-related DNA repair. One of these is the histone chaperone FACT complex subunit Spt16 that binds to RNF20 to enable recruitment of SNF2H as well as the DNA repair proteins BRCA1 and RAD51 at DSBs to initiate HRR [66]. RNF40 was shown to have a similar role and interaction with Spt16 at sites of DSBs [67]. A number of the H2Bub1 DUBs have also been implicated in the DNA damage response through modulation of chromatin, including USP11 [68], USP22 [69,70] and USP44 [71]. Furthermore, the DOT1L histone methyltransferase known to engage in trans-histone cross-talk with H2Bub1 is important for HRR at DSBs [72].

Global loss of H2Bub1 has been observed in cancer cell line models following treatment with DNA damaging agents including doxorubicin, neocarzinostatin and cisplatin [14,23,24]. On the background of this global loss, H2Bub1 is enriched in the coding regions of specific highly expressed genes, including p53 target genes such as *CDKN1A* and *MDM2* [23,24]. It is likely in these cases that many genes that display enriched H2Bub1 following DNA damage are required for decisions regarding cellular fate [23].

## 4. H2Bub1 and Cellular Proliferation in Cancer

It is well established that cellular proliferation is dependent upon access to chromatin to regulate the expression of oncogenes and tumour suppressors that when aberrantly expressed can lead to a malignant phenotype. Loss of H2Bub1 has been associated with tumour progression consistent with a tumour suppressive function of this histone PTM [1,10,12]. The role of H2Bub1 in cellular proliferation has been studied predominantly through manipulation of its key writer and eraser enzymes. For example, siRNA (short interfering RNA) down-regulation of RNF20 and RNF40 has been reported to inhibit proliferation of prostate cancer cells [42]. Of note, RNF40 has been implicated in the control of key apoptotic genes in colorectal cancer cells [73]. 

RNF20 has also been identified as necessary for proliferation, both in vitro in mixed-lineage leukemia (MLL)-rearranged human acute myeloid leukemia (AML) cell lines and in vivo, being important for disease progression in a genetically engineered mouse model of AML [74]. RNF20 and USP44 have different effects on the proliferation and migration of breast cancer cells dependent on subtype, i.e., basal-like versus luminal [22]. H2Bub1-associated DUBs USP22, USP51 and USP27X have been shown in vitro to be required for normal growth, with in vivo depletion suppressing tumour growth in a mouse xenograft model of breast cancer cells [75]. Down-regulation of USP36 in lung cancer cells has also been shown to negatively impact on cell proliferation [76]. A number of these associations are discussed elsewhere in this review.

## 5. H2Bub1 is a Key Regulator of Developmental Transitions

### 5.1. H2Bub1 and Stem Cell Plasticity

Histone PTMs are important for maintaining stem cell self-renewal and the plasticity of stem cells, as well as facilitates stem cell differentiation into different cell lineages [77,78,79,80,81]. H2Bub1 levels have been shown to increase during the differentiation of embryonic stem cells (ESCs) and embryonal carcinoma stem cells (ECSCs) [82], as well as in human mesenchymal stem cells (hMSCs) after differentiation into osteoblasts and adipocytes [83]. Numerous components of the H2Bub1 interactome have been implicated in the maintenance of stem cell pluripotency and differentiation, including the writers RNF20 [82] and RNF40 [83], cyclin-dependent kinase CDK9 [83], the BAF250b (BRG1/BRM-Associated Factor) subunit of the SWI/SNF chromatin remodelling complex [84], H2Bub1-interacting histone methyltransferase complexes including COMPASS [85] and DOT1L [86,87,88], as well as the DUBs USP22 [89,90,91,92] and USP44 [82,93]. As for DNA damage, H2Bub1 and many of its regulatory factors that modulate chromatin play a central role in determining cellular fate as part of key signalling required for differentiation. In the case of cancer stem cells that are linked with tumour initiation, relapse and drug resistance, targeting specific components of the H2Bub1 interactome may offer new therapeutic strategies for drug resistant malignancies [88,90,91,93].

### 5.2. H2Bub Regulates Developmental Pathways and the Immune Response in Plants

Drawing parallels between the human and the plant world, H2Bub1 and the E2 and E3 ligases involved in its writing have been shown to have major roles in the control of gene expression linked to developmental and immune functions in a range of plant species. E2 and E3 ligases that write H2Bub1 in *Arabidopsis thaliana* are pivotal in controlling expression of *FLOWERING LOCUS C* (*FLC*) that is the main repressor of flowering, thus dictating flowering time [25,94]. H2Bub1 in *Arabidopsis* has also been linked to the regulation of circadian clock genes [26]. Down-regulation of the E3 ligase encoding flowering-related RING Protein 1 (*FRRP1*) in rice, *Oryza sativa*, has been shown to regulate flowering time and yield potential, most likely through H2Bub1 [95]. Seed germination is another key developmental transition for plants. H2Bub1 controlled by E3 ligases has been shown to control the expression of genes involved in seed dormancy in *Arabidopsis thaliana* [27]. Further, variation in monoubiquitination of histone H2A and H2B variants has been linked to the innate immune response against fungal attack in rice, and H2Bub1 has been shown to have a role in defence against the fungus *Botrytis cinerea* in tomato, again via its E3 ubiquitin ligases [28,96]. 

Considered together, H2Bub1 has a significant role in the plant ubiquitin chromatin landscape with regulatory influence over the expression of genes in developmental programs including the circadian clock, flowering time, seed dormancy and germination, as well as in pathogen defence. The role of H2Bub1 spans multicellular eukaryotes and key cellular processes, establishing its importance as a major epigenomic regulator of gene expression in both the animal and plant kingdom. 

## 6. Global Loss of H2Bub1 in Human Malignancy

Global loss of H2Bub1 in primary tumours or early lesions has been detected using immunohistochemistry by us and others across a range of malignancies. H2Bub1, RNF20 and/or RNF40 have been investigated in numerous cancer tissues, including breast [22,40,41,97,98], ovarian [62,99], colorectal [21,97,100], lung [97,101], gastric [102], kidney [103] and parathyroid [18]. Where clear numbers have been reported in publications, we have summarised this data; for H2Bub1 (Table 1) and RNF20/RNF40 (Table 2). In some cancers, H2Bub1 loss by way of loss of RNF20 has been implicated in an early inflammatory response, while in others it has been linked to progression of the tumour and in some cases, a worse prognosis. 

### 6.1. H2Bub1 Loss is Associated with an Inflammatory Response that may Increase Risk of Developing Cancer

The ability to mount an appropriate inflammatory response is part of our key defence mechanisms against pathogens and disease; however, it is when inflammation becomes chronic that problems can arise, including acting as a trigger for tumorigenesis [105]. Chronic inflammation, such as that seen in inflammatory bowel disease (IBD) and ulcerative colitis (UC), is driven by inflammatory mediators such as nuclear factor (NF)-κB and signal transducer and activator of transcription 3 (STAT3), cytokines such as interleukin 6 (IL-6) and prostaglandins, and has been linked to an increased risk of developing cancer [106,107,108,109,110]. Using a model of *Rnf20* heterozygous mice to drive depleted H2Bub1, as well as human samples of UC and colorectal cancer, Tarcic and colleagues have shown that loss of H2Bub1 creates a tumour promoting microenvironment in the colon centred on the chronic inflammatory response [21]. The mechanism of up-regulation of genes associated with inflammation such as IL-6 and IL-8 in conjunction with depleted H2Bub1 is through the pro-inflammatory cytokine tumour necrosis factor alpha (TNF-α), itself mediated through NF-κB activation [110]. In non-cancerous epithelial cells in this model, depletion of *Rnf20* and as a result H2Bub1, fostered a pro-inflammatory transcriptional response. Furthermore, gene expression of both *RNF20* and *RNF40*, along with down-regulation of H2Bub1, was identified in epithelial and stromal colonic tissue from patients with UC and was inversely correlated with inflammatory cytokines *IL6* and *IL8* [21]. 

Studies comparing basal-like cells of triple negative breast cancer (TNBC) that are aggressive and poorly differentiated tumours, with breast tumours of luminal origin with a more favourable prognosis showed that TNBCs had expression of a greater number of inflammatory genes, including *IL6* and *IL8* [22,111]. Down-regulation of *RNF20* further increased the expression of *IL6* and *IL8* via NF-κB signalling in basal-like breast cancer cells and depleted the repressive chromatin mark H3K9me3 at their promoters [22]. In luminal breast cancer cells, however, *RNF20* down-regulation was associated with a decrease in expression of ER gene targets that drive proliferation and migration including *PGR*, *CXCL12* and *FOXA1* [22]. This finding goes some way towards explaining the apparent opposite roles of RNF20 and H2Bub1 in basal and luminal breast cancers, whereby RNF20 and H2Bub1 appear to function in a tumour-suppressive role in basal-like breast cancer cells and be pro-oncogenic in luminal breast cancer cells, driving tumorigenesis. Further supporting this phenomenon of a breast cancer subtype-specific function of RNF40 and H2Bub1, Wegwitz and colleagues have shown that the RNF40-H2Bub1 axis actually supports growth of HER2-positive (HER2+) breast cancers, both in vitro and in vivo, via transcriptional activation of genes involved in maintenance of the actin cytoskeleton. This important work highlights the opportunity for therapeutic intervention that targets RNF40 and/or H2Bub1 in the HER2+ breast cancer subtype [112].

In addition to discoveries linking H2Bub1 and its ligation machinery to inflammatory conditions of the colon with an increased risk of malignancy and developing TNBC, Hooda and colleagues discovered modulation of H2Bub1 levels in pre-cursor cells of ovarian cancer with links to inflammatory genes [62]. Here, upon depletion of *RNF20* and concomitant loss of H2Bub1 in fallopian tube epithelial cells, the presumed site of origin of many high-grade serous ovarian cancers (HGSOCs), IL6 was elevated and enhanced migration observed [62]. It is likely that additional tumour cell types will be discovered to increase the production of inflammatory cytokines in response to depletion of RNF20 and H2Bub1, contributing to a microenvironment conducive to initiating malignancy and/or driving tumorigenesis. 

### 6.2. Global Loss of H2Bub1 Is an Early Event in Some Cancers

Two studies support that H2Bub1 loss is a very early event in ovarian tumorigenesis, with loss detected in serous tubal intraepithelial carcinomas (STICs) now known to be the precursor lesion for a large proportion of HGSOC [62,113,114], and also equally across all stages (I-IV) of HGSOC [99]. Dickson and colleagues demonstrated global loss of nuclear H2Bub1 in 77% (313 of 407) of HGSOC [99]. No correlation was made between loss of H2Bub1 and either progression free or overall survival. Furthermore, no correlation was detected between the presence of a *BRCA1* or *BRCA2* mutation and H2Bub1 loss. In the same tumour cohort, complete loss of RNF20 assessed using immunohistochemical staining was seen in only 6% of tumours (26 of 424) and did not correlate with loss of H2Bub1, implying that abrogation of RNF20 function does not explain the majority of H2Bub1 loss present in these tumours. Of note, no HGSOC had both a *BRCA1* mutation and RNF20 loss, suggesting that functional abrogation of both of these E3 ligases in the same cell may be lethal [99]. 

In apparent contrast to this earlier study of RNF20 immunohistochemistry in HGSOC, Hooda and colleagues reported that over half of HGSOC in The Cancer Genome Atlas (TCGA; *n* = 579) are heterozygous for *RNF20* and that 36% were heterozygous for *RNF40* [62]. RNF20 protein expression would need to be interrogated in TCGA HGSOC samples to definitively correlate RNF20 and H2Bub1 in this cohort. A complete picture of the factors responsible for the maintenance of H2Bub1 in ovarian and fallopian tube epithelia remains to be elucidated; however, it would appear that H2Bub1 E3 ubiquitin ligase writers, of which there are multiple [1,10] (Figure 1), can functionally substitute for one another in some circumstances. Levels of RNF20 and RNF40 have also been reported to be lower than normal tissue in testicular seminomas, with the suggestion that their loss occurs prior to development of the invasive phenotype [115]. 

### 6.3. Global Loss of H2Bub1 Is Linked to Tumour Progression and a Worse Prognosis

One of the earliest immunohistochemical studies of global H2Bub1 levels in primary tumours was conducted in breast cancer, where a clear increase in H2Bub1 loss was observed over tumour progression, from no loss in normal adjacent tissue and benign tumours, to 67% loss in malignant tumours and 84% loss in breast cancer metastatic deposits [40]. While this study did not analyse tumours based on sub-type, Tarcic and colleagues have subsequently investigated H2Bub1 and its ligation machinery in basal-like (TNBC) and luminal breast cancers [22]. Here, the correlation between H2Bub1 levels and patient outcome was dependent on breast tumour subtype. For women with breast tumours that were ER positive (ER+) and of a luminal subtype, higher levels of H2Bub1 correlated with shorter survival times, while in TNBCs of a basal-like subtype, higher levels of H2Bub1were associated with longer survival [22]. Basal-like tumours had lower H2Bub1 levels relative to other breast sub-types, and are well known to have a worse prognosis. As already noted, in vitro experiments interrogating cell migration and proliferation showed that depleting RNF20 that reduced H2Bub1 levels, acted to increase proliferation and migration of basal-like breast cancer cells, yet decreased these functional endpoints in luminal-type cells. These growth patterns were recapitulated in a mouse model, whereby down-regulation of RNF20 in basal-like cells resulted in faster growing mammary tumours relative to control cells, while down-regulation of RNF20 in luminal type cells led to slower growth than control cells [22]. Further in these preclinical models, metastases to the lung reflected the rate of tumour growth, with down-regulation of RNF20 resulting in an increase of lung metastases from basal-like cells only. Therefore at least in breast cancer, the role of RNF20 and H2Bub1 is highly dependent on cell type of origin; in fact this role is dichotomous in basal-like versus luminal breast cancer cells. 

H2Bub1 loss has been strongly associated with a worse prognosis in colorectal cancer independent of nodal stage; however, loss of H2Bub1 alone was not an independent prognostic marker [100]. Patients with gastric cancer that stain positively for H2Bub1 had a higher 5 year survival compared to patients where H2Bub1 was lost; in fact, H2Bub1 was an independent prognostic factor for survival in this cancer type [102]. Here, loss of H2Bub1 was increasingly associated with more poorly differentiated tumours [102]. This was also the case for lung tumours and further, lung cancer patients whose tumours were positive for H2Bub1 displayed a trend towards increased survival compared with patients whose tumours had lost H2Bub1 [101]. Decreasing levels of tumour differentiation in parallel with loss of H2Bub1 has also been reported in breast tumours, consistent with a role for H2Bub1 in maintaining a differentiated phenotype [41]. Hahn and colleagues demonstrated global loss of H2Bub1 in familial and sporadic parathyroid tumours associated with mutation of a gene encoding a member of the PAF1C, *CDC73* [18]. Benign parathyroid tumours with wild-type *CDC73* did not demonstrate loss of H2Bub1. 

## 7. Cancer-Related Proteins and the H2Bub1 Interactome

The interplay between complexes and factors that regulate the epigenome and cancer-related proteins is important for chromatin modelling and gene expression. Understanding this interplay, both in healthy cells and in malignancy where tumour suppressors are frequently mutant and/or silenced is an emerging and intricate field. There is a growing list of cancer-related proteins, many of which have a tumour suppressive function, that either interact with, or constitute the ubiquitin ligation machinery of H2Bub1, including RNF20 and RNF40, p53, BRCA1, CDC73, members of the SWI/SNF chromatin remodelling complex, members of histone methyltransferase complexes involved in cross-talk with H2Bub1 including DOT1L and COMPASS, and numerous deubiquitinases including USP22 and USP44 (Figure 1). Below we discuss a number of these interactors, including their genetic or epigenetic abrogation in malignancy, and acknowledge that there are likely many more that remain to be discovered. 

### 7.1. RNF20 and RNF40 E3 Ubiquitin Ligases

The majority of reports in the literature describe RNF20 in a manner consistent with being classified as a tumour suppressor; however, some reports suggest a pro-oncogenic activity. While the RNF20/RNF40 complex is accepted as the main E3 ligase complex responsible for writing H2Bub1, substrates other than K120 on histone H2B have been reported. For instance, RNF20 polyubiquitinates the ErbB3 receptor binding protein Ebp1 [116] and ZSCAN4 (Zinc Finger and SCAN Domain Containing 4) that is involved in telomere maintenance, genomic stability and mouse embryonic stem cells [117]. The RNF20/RNF40 complex can also monoubiquitinate the motor protein Eg5, a protein belonging to the kinesin-like family with roles in spindle dynamics and assembly associated with mitosis [118]. The rat orthologue of RNF40, Staring, polyubiquitinates the nervous system specific protein Syntaxin 1 marking it for proteasomal degradation [119].

In line with a tumour suppressive function, copy number loss of *RNF20* has been reported in HGSOC [62] and pre-invasive dysplastic airway lesions [120]. A low frequency of *RNF20* and *RNF40* mutations have been reported in colorectal cancer [121,122]. *RNF20* and/or *RNF40* transcript levels are depleted in a number of malignant or pre-malignant tissues, including in colonic tissue from patients with UC [21], in metastatic prostate cancer cells when compared with benign disease [123] and is lower in testicular germ cell cancer seminoma relative to normal testis [115]. Hypermethylation of the *RNF20* promoter has also been discovered in primary breast cancers, consistent with a tumour suppressive role for RNF20 [98]. Indirectly, RNF20 may function in a tumour suppressive capacity to obstruct the expression of oncogenes including *MYC* and *FOS* located in regions of compacted chromatin by interfering with recruitment of TFIIS that would usually function to relieve stalled RNA pol II [60]. RNF20 does this by obstructing the interaction between TFIIS and PAF1C. 

In apparent contrast and already discussed in this review, a pro-oncogenic role for RNF20 has been reported in breast cancer cells of luminal, but not basal cell-type origin [22]. RNF20 also has an oncogenic role in MLL fusions that drive aberrant gene expression in haematopoietic cells and are initiators of leukemogenesis. H2Bub1 enrichment correlating with transcriptional elongation is observed in MLL-fusion target genes [74]. Furthermore, in colorectal cancer, RNF40 has been reported to promote inflammatory signalling through NF-κB signalling [124] and in hepatocellular carcinoma, RNF40 has been shown in a large cohort (*n* = 130) to be almost equally expressed at high or low levels, with higher levels correlating with a worse prognosis [104].

Considering more broadly the cellular turnover of RNF20 and the role of additional factors, the HECT-domain E3 ubiquitin ligase Smad ubiquitin regulatory factor 2 (Smurf2) that itself has tumour suppressive roles, polyubiquitinates RNF20, marking it for proteasomal degradation, and in this way is linked in with the regulation of H2Bub1 [125]. The relationship between Smurf2 and RNF20 is likely the mechanism that accounts for the role of Smurf2 in regulating the chromatin landscape and maintaining genomic stability [126]. It is also involved in determining levels of RNF20 available to participate at sites of DSBs as part of the DDR [125].

RNF40 also has interaction partners, an important one being the histone chaperone human Suppressor of Ty Homologue-6 (SUPT6H) that likely works in a functional complex with RNF40 and RNF20 to control H2Bub1 levels [41]. All of SUPT6H, RNF20, RNF40 and ERα have been shown to form a complex together at phosphorylated Ser 2 on the carboxy-terminal domain (CTD) of RNA Pol II [41]. This relationship provides a platform for estrogen modulation of the chromatin landscape, driving transcriptional elongation of ERα target genes important for proliferation and differentiation of the mammary epithelium. Like H2Bub1 levels, SUPT6H levels were shown to decrease over the course of breast tumorigenesis, with more malignant breast tumours with a worse prognosis demonstrating less SUPT6H and H2Bub1 [41]. In addition to association with ERα and regulation of ER target genes, RNF20 and RNF40 have been shown to physically associate with the AR, with the H2Bub1/RNF20/RNF40 axis implicated in AR-associated gene transcription and affecting the growth of prostate cancer cells [42]. Of note, both RNF20 and RNF40 have been linked to the maintenance of genomic stability [104,115,125,127].

### 7.2. p53 Associates with the E3 Ubiquitin Ligase RNF20

The tumour suppressor p53 is encoded by the most frequently mutated gene in human malignancy, being mutated in over 50% of all cancers, and referred to as the guardian of both the genome and the epigenome [128,129]. p53 has been shown to directly interact with RNF20 and furthermore, RNF20 and/or the RNF20/RNF40 complex have been identified as transcriptional co-activators at the promoters of p53 target genes including *CDKN1A* (encoding p21), *MDM2*, and *BBC3* (encoding PUMA) [17,130]. Loss of the RNF20/RNF40 complex correlated with depleted levels of H2Bub1 in the coding regions of these genes and reduced transcript levels [24,130]. A recent study investigating H2Bub1 enrichment at p53 target genes in p53 wild-type and gain-of-function (GOF) mutant cell line models showed absence of enrichment in the presence of *TP53* mutations [23]. It will be interesting to determine whether GOF mutant p53 cells can drive tumorigenesis by mechanisms such as H2Bub1 enrichment and correlated increased gene expression at GOF mutant p53 target genes. 

Furthermore, while the oncoprotein Human Double Minute 2 (HDM2), also frequently referred to in the literature as MDM2, is well known as both a p53 target gene and the RING domain E3 ubiquitin ligase that polyubiquitinates p53 for degradation via the proteasome [131], it has also been reported to function as an enzymatic writer for H2Bub1 [132]. This would appear to be; however, on free histone H2B, and not when H2B is part of the native nucleosome core where the RNF20/RNF40 complex dominates as the main E3 ubiquitin ligase for H2Bub1 [12,133].

### 7.3. CDC73 Is a Binding Partner of RNF20 and RNF40

CDC73 (Cell Division Cycle 73; also known as parafibromin) is a classic tumour suppressor and core member of the human PAF1C [13]. It is also present in *Drosophila* (dCdc73) where it is more commonly referred to as Hyrax [134] and in yeast (yCdc73) [135]. In humans, mutations in *CDC73* (also known as *HRPT2*, Hyperparathyroidism 2) are present in germline DNA of patients with the inherited conditions Hyperparathyroidism Jaw Tumour Syndrome (HPT-JT; OMIM 145001) and Familial Hyperparathyroidism (FIHP; OMIM 145000) [136] reviewed in [137]. Somatic *CDC73* mutations are also observed in parathyroid carcinoma [138,139]. Surrogate detection of *CDC73* mutations by nuclear loss of immunohistochemical staining for CDC73 (parafibromin) in parathyroid neoplasms is a recognised diagnostic for HPT-JT and/or parathyroid cancer [140,141,142,143,144]. 

Using a yeast two-hybrid assay, CDC73 was shown to directly bind to both E3 ubiquitin ligases RNF20 and RNF40 [18]. Furthermore, loss of nuclear CDC73 in primary parathyroid tumours as the result of *CDC73* mutation led to significant depletion of H2Bub1, suggesting that CDC73 functions as a key protein associating the PAF1C with the ligase machinery that synthesises H2Bub1 [18]. It is likely that global loss of H2Bub1 and the entailing abrogation of gene expression is a major mechanism by which mutant CDC73 exerts its tumorigenic effect. 

### 7.4. BRCA1-BARD1 Is an E3 Ubiquitin Ligase for H2Bub1

Germline mutations in *BRCA1* are well known to increase the risk of developing breast and/or ovarian cancer, with somatic mutations of this tumour suppressor also identified in sporadic tumours [145,146,147,148,149,150,151]. The BRCA1 Associated RING Domain 1 (BRCA1-BARD1) heterodimer is one of a number of E3 ligase complexes reported to write H2Bub1 [19]. In fact, BRCA1-BARD1 has been shown to ubiquitinate all core histones and the histone variant H2AX [152]. Despite this clear function of the BRCA1-BARD1 complex as a writer of H2Bub1, a large study previously discussed in this review that analysed primary HGSOC characterised for *BRCA1* mutation was unable to demonstrate any correlation between *BRCA1* mutation and loss of H2Bub1 [99]. Given that there are multiple E3 ligases/ligase complexes that write H2Bub1, it is likely that these enzymes may be able to work in concert to compensate for loss of one in order to maintain this important histone modification. 

### 7.5. SWI/SNF Chromatin Remodelling Complexes, Including ARID1B/BAF250b

Initially discovered in yeast, the SWI/SNF complexes are members of the family of adenosine triphosphate (ATP) dependent chromatin remodelling complexes [153,154]. There are almost 30 known genes encoding subunits of the SWI/SNF complexes, comprised of three distinct complexes with unique and overlapping subunits (canonical BAF (cBAF), polybromo-associated BAF (PBAF) and non-canonical BAF (ncBAF) (reviewed in [155]). Around 20% of all human malignancies harbour a mutation in a gene encoding a SWI/SNF complex member [155,156]. Members of the SWI/SNF complex, including BAF155, BAF170, BRG1 and BRM were all shown to associate with H2Bub1 enriched chromatin [20]. Furthermore, SWI/SNF was shown to be required for optimal transcriptional elongation for genes reliant on RNF20 and H2Bub1 for their expression [20]. In this way, H2Bub1 appears to act as a recruitment scaffold or docking platform for the SWI/SNF chromatin remodelling complex to ensure optimal gene expression. Further demonstrating a key relationship between SWI/SNF and H2Bub1 is the discovery that the SWI/SNF complex member BAF250b/ARID1b form part of an E3 ubiquitin ligase complex along with elongin C (Elo C), Cullin-2 and Roc-1 to write H2Bub1 [157].

### 7.6. H2Bub1 Deubiquitinases—USP22 and USP44

Ubiquitin is erased from histone H2B by DUBs of which there are at least twelve, not all in the mammalian setting, reported in the USP family that can perform this function—USP3 [158], USP7 [159], USP11 [68], USP12 [160], USP15 [161], USP22 [162], USP27X [75], USP36 [76], USP44 [82,163], USP46 [160], USP49 [164] and USP51 [75]. Many of these DUBs have previously been reviewed in the context of erasing H2Bub1 [1,10,12], therefore the focus here will be on recent discoveries, and specifically on USP22 and USP44. 

USP22 is perhaps the most well studied of the H2Bub1-associated DUBs [165]. It has been implicated as part of the “death-from-cancer” signature, a stem cell gene expression signature consisting of 11 genes prognostic of rapid relapse and resistance to therapy in different solid tumours [92,166]. Expression of USP22 has been associated with stemness in cancer. In addition to its links with H2Bub1, USP22 also has non-histone substrates with important links to cancer. One of these is with the oncogene c-Myc where USP22 has been reported to function as a DUB to increase c-Myc stability in breast cancer cell lines with implications for the progression of this malignancy [167]. USP22 also been shown to deubiquitinate the transcriptional regulator far upstream element (FUSE)-binding protein 1 (FBP1), directly affecting the expression of p21 and impacting upon cell proliferation and tumorigenesis [168].

USP22 functions as a subunit of the SAGA chromatin modifying complex that is a complex capable of mediating deubiquitination and acetylation of histone and non-histone substrates [162,169]. The deubiquitinating module within SAGA consists of USP22, ATXN7L3, ATXN7 and ENY2 [165,170]. In humans, this deubiquitinating SAGA module is co-located with H2Bub1 within actively transcribed genes and is classified as a global transcriptional activator important for all RNA Pol II transcription [171,172]. USP22 has been associated with increased angiogenesis, cancer cell proliferation and metastasis, cisplatin and irradiation resistance and DNA damage signalling [173]. Targeting of USP22 is being explored as a cancer therapeutic [90,173]. Of particular interest, USP22 has recently been implicated as a regulator of PD-L1, marking it as a potential key factor in immune evasion that facilitates tumorigenesis [174]. Additional evidence that USP22 is involved in the immune response is its opposing role with RNF20 in determining expression of the transcription factor FOXP3 (Forkhead box protein P3) that determines the development and function of regulatory T (T_reg_) cells that are important for the maintenance of self-tolerance and immune homeostasis [175].

Of note, USP44, co-operating with USP7, has also been identified as a DUB for FOXP3, linking these DUBs to T_reg_ cells and the immune response and flagging them as possible therapeutic targets for malignancy [176]. Furthermore, USP44 has recently been implicated in the innate immune response mounted against DNA viruses as a positive regulator of the Stimulator of Interferon Genes (STING) protein [177]. USP7 has also been implicated in destabilisation of the HDM2/p53 axis that is fundamentally important in malignancy [178]. 

In basal-like TNBCs, there is an inverse relationship between USP44 and RNF20/RNF40 with USP44 being more highly expressed, concomitant with lower levels of H2Bub1 in these tumours [22]. In cell line models of basal-like TNBC, down-regulation of USP44 leading to higher levels of H2Bub1 showed decreased proliferation and migration; however, the opposite was true for proliferation in luminal cell line models, highlighting the importance of consideration of cell type when determining therapeutic targeting of the H2Bub1 interactome [22]. As noted earlier, USP44 has been identified at sites of DSBs [71].

## 8. Conclusions

H2Bub1 is a key histone modification influencing fundamental cellular activities including transcriptional elongation, the DDR and stem cell plasticity. It is increasingly recognised as a central histone PTM given its influence and/or interactions with other chromatin remodelling complexes such as COMPASS, DOT1L, SAGA and SWI/SNF. The association of H2Bub1 with a growing list of cancer-related proteins affirms its clear connection to cancer. Changes in H2Bub1 enrichment levels have been implicated at all stages of tumour progression, from early lesions to metastatic disease, including association with inflammation that may increase the risk of developing malignancy in some tissues. 

The importance of H2Bub1 and its modifying enzymes is now clearly established in both normal cellular processes and in malignancy, yet we do not have clear explanations regarding some apparent anomalies. Enrichment of H2Bub1, or lack thereof, in gene bodies does not always correlate with gene expression. In fact depletion of the main H2Bub1 writers RNF20 and RNF40 only influences the expression of a subset of genes [61,98]. This may be explained by selective regulation based on H2Bub1 levels, with only those genes with low to moderate H2Bub1 enrichment affected [61]. The high level of degeneracy or compensatory functions offered by multiple H2Bub1 writers and erasers likely also plays a role in maintaining the necessary levels of H2Bub1 enrichment for the maintenance of healthy tissue and during development.

Stimulus-specific gene expression, such as seen in p53 target genes in response to DNA damage, appear to have a strong correlation with H2Bub1 enrichment [23,24]. This would also seem to be true for hormonally stimulated gene expression, including genes relying on the transcription factors ERα and the AR [40,42]. The dynamic nature of histone PTMs, and specifically H2Bub1 in rapid chromatin remodelling, is likely crucial for the high-speed responses needed for expression of many of these genes. This is likely also true in developmental programming, including in the differentiation of mesenchymal and embryonic stem cells. Yet another complexity of H2Bub1 and its associated writers and erasers discussed in this review is seen in the context of different cellular subtypes of malignancy, with clear examples of opposing proliferative and migratory effects of manipulating H2Bub1 ligation machinery in luminal versus basal-like breast cancers [22]. It would appear that H2Bub1 and its associated factors can function as drivers of tissue specific transcription patterns associated with distinct cellular types.

Taken together, it is clear that the complexities of H2Bub1 and its associated machinery must be considered in a context specific fashion. Continued expansion and elucidation of the H2Bub1 interactome offers new insights into cellular processes and extends our opportunities for therapeutic targeting of malignancy based on the epigenome and ubiquitin chromatin modelling.

## Figures and Tables

**Table 1:** H2Bub1 loss in primary tumours.

Tissue Type	Cohort Size	% H2Bub1 Loss	Reference
Breast—normal mammary duct epithelial cells (adjacent tissue)	8	0%	[40]
Breast—benign tumour	18	0%	[40]
Breast—cancer	64	67%	[40]
Breast—cancer	34	97%	[97]
Colon—normal mucosa	55	0%	[100]
Colon—cancer	36	86%	[97]
Colon—cancer	1584	21% ^^^	[100]
		26% ^^^^	
Gastric cancer (well differentiated)	23	4% ^^^	[102]
		30% ^^^^	
Gastric cancer (moderately differentiated)	55	13% ^^^	[102]
		40% ^^^^	
Gastric cancer (poorly differentiated)	81	21% ^^^	[102]
		77% ^^^^	
Lung—cancer	36	97%	[97]
Lung—adenocarcinoma (well differentiated)	28	31%	[101]
Lung—adenocarcinoma (moderately differentiated)	76	46%	[101]
Lung adenocarcinoma—(poorly differentiated)	59	54%	[101]
HGSOC	407	77% ^^^	[99]
		19% ^^^^	
HGSOC	18	44% ^^^	[62]
		56% ^^^^	
Fallopian tube STIC	25	24% ^^^	[62]
		76% ^^^^	
Normal FTE	23	9% ^^^	[62]
		74% ^^^^	
Parathyroid tumours (*CDC73*-associated)	11	55% ^^^	[18]
		45% ^^^^	

Where H2Bub1 data was reported both as total loss or weak to moderate immunohistochemical levels, it is identified as follows: ^^^ total loss of H2Bub1 (no nuclear H2Bub1 present detected by immunohistochemistry); ^^^^ weak to moderate H2Bub1 detected by immunohistochemistry. HGSOC, High-grade serous ovarian cancer; STIC, Serous Tubal Intraepithelial Carcinoma; FTE, fallopian tube epithelium.

**Table 2:** RNF20 and RNF40 loss in primary tumours.

**Tumour Type**	**Cohort Size**	**RNF20** ^^^	**RNF20** ^^^^	**% Tumours** **with RNF20** **Loss**	**Reference**
HGSOC	424	√		6% ^^^^^	[99]
				7% ^^^^^^	
HGSOC	579		√	53%	[62]
Lung adenocarcinoma	517		√	~25% *	[101]
**Tumour Type**	**Cohort Size**	**RNF40** ^^^	**RNF40** ^^^^	**% Tumours** **with RNF40** **Loss**	**Reference**
HGSOC	579		√	36%	[62]
HCC	130	√		50% **	[104]

^^^ protein levels assessed by immunohistochemistry; ^^^^ gene transcript levels; ^^^^^ total loss (immunohistochemical score of 0); ^^^^^^ intermediate staining; * refers to lower RNF20 levels in malignant lung tissue versus normal lung tissue. ** the remainder of this cohort was described as staining high for RNF40. HGSOC, High-grade serous ovarian cancer; HCC, hepatocellular carcinoma.

## References

[1] Fuchs G., Oren M. (2014). Writing and reading H2B monoubiquitylation. Biochim. Biophys. Acta.

[2] Dawson M.A., Kouzarides T., Huntly B.J. (2012). Targeting epigenetic readers in cancer. N. Engl. J. Med..

[3] Dawson M.A., Kouzarides T. (2012). Cancer epigenetics: From mechanism to therapy. Cell.

[4] Marsh D.J., Dickson K.A. (2019). Writing histone monoubiquitination in human malignancy—The role of RING finger E3 ubiquitin ligases. Genes.

[5] Margueron R., Reinberg D. (2011). The Polycomb complex PRC2 and its mark in life. Nature.

[6] Fang J., Chen T., Chadwick B., Li E., Zhang Y. (2004). Ring1b-mediated H2A ubiquitination associates with inactive X chromosomes and is involved in initiation of X inactivation. J. Biol. Chem..

[7] Lipkowitz S., Weissman A.M. (2011). RINGs of good and evil: RING finger ubiquitin ligases at the crossroads of tumour suppression and oncogenesis. Nat. Rev. Cancer.

[8] Deshaies R.J., Joazeiro C.A. (2009). RING domain E3 ubiquitin ligases. Annu. Rev. Biochem..

[9] Budhidarmo R., Nakatani Y., Day C.L. (2012). RINGs hold the key to ubiquitin transfer. Trends Biochem. Sci..

[10] Cole A.J., Clifton-Bligh R., Marsh D.J. (2015). Histone H2B monoubiquitination: Roles to play in human malignancy. Endocr.-Relat. Cancer.

[11] Cao J., Yan Q. (2012). Histone ubiquitination and deubiquitination in transcription, DNA damage response, and cancer. Front. Oncol..

[12] Johnsen S.A. (2012). The enigmatic role of H2Bub1 in cancer. FEBS Lett..

[13] Jaehning J.A. (2010). The Paf1 complex: Platform or player in RNA polymerase II transcription?. Biochim. Biophys Acta..

[14] Moyal L., Lerenthal Y., Gana-Weisz M., Mass G., So S., Wang S.Y., Eppink B., Chung Y.M., Shalev G., Shema E. (2011). Requirement of ATM-dependent monoubiquitylation of histone H2B for timely repair of DNA double-strand breaks. Mol. Cell.

[15] Trujillo K.M., Osley M.A. (2012). A role for H2B ubiquitylation in DNA replication. Mol. Cell.

[16] Sadeghi L., Siggens L., Svensson J.P., Ekwall K. (2014). Centromeric histone H2B monoubiquitination promotes noncoding transcription and chromatin integrity. Nat. Struct. Mol. Biol..

[17] Kim J., Hake S.B., Roeder R.G. (2005). The human homolog of yeast BRE1 functions as a transcriptional coactivator through direct activator interactions. Mol. Cell.

[18] Hahn M.A., Dickson K.A., Jackson S., Clarkson A., Gill A.J., Marsh D.J. (2012). The tumor suppressor CDC73 interacts with the ring finger proteins RNF20 and RNF40 and is required for the maintenance of histone 2B monoubiquitination. Hum. Mol. Genet..

[19] Thakar A., Parvin J., Zlatanova J. (2010). BRCA1/BARD1 E3 ubiquitin ligase can modify histones H2A and H2B in the nucleosome particle. J. Biomol. Struct. Dyn..

[20] Shema-Yaacoby E., Nikolov M., Haj-Yahya M., Siman P., Allemand E., Yamaguchi Y., Muchardt C., Urlaub H., Brik A., Oren M. (2013). Systematic identification of proteins binding to chromatin-embedded ubiquitylated H2B reveals recruitment of SWI/SNF to regulate transcription. Cell Rep..

[21] Tarcic O., Pateras I.S., Cooks T., Shema E., Kanterman J., Ashkenazi H., Boocholez H., Hubert A., Rotkopf R., Baniyash M. (2016). RNF20 Links Histone H2B Ubiquitylation with Inflammation and Inflammation-Associated Cancer. Cell Rep..

[22] Tarcic O., Granit R.Z., Pateras I.S., Masury H., Maly B., Zwang Y., Yarden Y., Gorgoulis V.G., Pikarsky E., Ben-Porath I. (2017). RNF20 and histone H2B ubiquitylation exert opposing effects in Basal-Like versus luminal breast cancer. Cell Death Differ..

[23] Cole A.J., Dickson K.A., Liddle C., Stirzaker C., Shah J.S., Clifton-Bligh R., Marsh D.J. (2020). Ubiquitin chromatin remodelling after DNA damage is associated with the expression of key cancer genes and pathways. Cell. Mol. Life Sci..

[24] Minsky N., Shema E., Field Y., Schuster M., Segal E., Oren M. (2008). Monoubiquitinated H2B is associated with the transcribed region of highly expressed genes in human cells. Nat. Cell Biol..

[25] Cao Y., Dai Y., Cui S., Ma L. (2008). Histone H2B monoubiquitination in the chromatin of FLOWERING LOCUS C regulates flowering time in Arabidopsis. Plant. Cell.

[26] Woloszynska M., Le Gall S., Neyt P., Boccardi T.M., Grasser M., Längst G., Aesaert S., Coussens G., Dhondt S., Van De Slijke E. (2019). Histone 2B monoubiquitination complex integrates transcript elongation with RNA processing at circadian clock and flowering regulators. Proc. Natl. Acad. Sci. USA.

[27] Liu Y., Koornneef M., Soppe W.J. (2007). The absence of histone H2B monoubiquitination in the Arabidopsis hub1 (rdo4) mutant reveals a role for chromatin remodeling in seed dormancy. Plant Cell.

[28] Zhang Y., Li D., Zhang H., Hong Y., Huang L., Liu S., Li X., Ouyang Z., Song F. (2015). Tomato histone H2B monoubiquitination enzymes SlHUB1 and SlHUB2 contribute to disease resistance against Botrytis cinerea through modulating the balance between SA- and JA/ET-mediated signaling pathways. BMC Plant Biol..

[29] Gatta R., Dolfini D., Zambelli F., Imbriano C., Pavesi G., Mantovani R. (2011). An acetylation-mono-ubiquitination switch on lysine 120 of H2B. Epigenetics.

[30] Fierz B., Chatterjee C., McGinty R.K., Bar-Dagan M., Raleigh D.P., Muir T.W. (2011). Histone H2B ubiquitylation disrupts local and higher-order chromatin compaction. Nat. Chem. Biol..

[31] Chandrasekharan M.B., Huang F., Sun Z.W. (2009). Ubiquitination of histone H2B regulates chromatin dynamics by enhancing nucleosome stability. Proc. Natl. Acad. Sci. USA.

[32] Fierz B., Kilic S., Hieb A.R., Luger K., Muir T.W. (2012). Stability of nucleosomes containing homogenously ubiquitylated H2A and H2B prepared using semisynthesis. J. Am. Chem. Soc..

[33] Paparidis N.F., Durvale M.C., Canduri F. (2017). The emerging picture of CDK9/P-TEFb: More than 20 years of advances since PITALRE. Mol. Biosyst..

[34] Pirngruber J., Shchebet A., Schreiber L., Shema E., Minsky N., Chapman R.D., Eick D., Aylon Y., Oren M., Johnsen S.A. (2009). CDK9 directs H2B monoubiquitination and controls replication-dependent histone mRNA 3’-end processing. EMBO Rep..

[35] Zhang F., Yu X. (2011). WAC, a functional partner of RNF20/40, regulates histone H2B ubiquitination and gene transcription. Mol. Cell.

[36] Sansó M., Lee K.M., Viladevall L., Jacques P., Pagé V., Nagy S., Racine A., St Amour C.V., Zhang C., Shokat K.M. (2012). A positive feedback loop links opposing functions of P-TEFb/Cdk9 and histone H2B ubiquitylation to regulate transcript elongation in fission yeast. PLoS Genet..

[37] Kwak H., Lis J.T. (2013). Control of transcriptional elongation. Annu. Rev. Genet..

[38] Sansó M., Parua P.K., Pinto D., Svensson J.P., Pagé V., Bitton D.A., MacKinnon S., Garcia P., Hidalgo E., Bähler J. (2020). Cdk9 and H2Bub1 signal to Clr6-CII/Rpd3S to suppress aberrant antisense transcription. Nucleic Acids Res..

[39] Kim J., Guermah M., McGinty R.K., Lee J.S., Tang Z., Milne T.A., Shilatifard A., Muir T.W., Roeder R.G. (2009). RAD6-Mediated transcription-coupled H2B ubiquitylation directly stimulates H3K4 methylation in human cells. Cell.

[40] Prenzel T., Begus-Nahrmann Y., Kramer F., Hennion M., Hsu C., Gorsler T., Hintermair C., Eick D., Kremmer E., Simons M. (2011). Estrogen-dependent gene transcription in human breast cancer cells relies upon proteasome-dependent monoubiquitination of histone H2B. Cancer Res..

[41] Bedi U., Scheel A.H., Hennion M., Begus-Nahrmann Y., Rüschoff J., Johnsen S.A. (2015). SUPT6H controls estrogen receptor activity and cellular differentiation by multiple epigenomic mechanisms. Oncogene.

[42] Jääskeläinen T., Makkonen H., Visakorpi T., Kim J., Roeder R.G., Palvimo J.J. (2012). Histone H2B ubiquitin ligases RNF20 and RNF40 in androgen signaling and prostate cancer cell growth. Mol. Cell. Endocrinol..

[43] Pavri R., Zhu B., Li G., Trojer P., Mandal S., Shilatifard A., Reinberg D. (2006). Histone H2B monoubiquitination functions cooperatively with FACT to regulate elongation by RNA polymerase II. Cell.

[44] Fleming A.B., Kao C.F., Hillyer C., Pikaart M., Osley M.A. (2008). H2B ubiquitylation plays a role in nucleosome dynamics during transcription elongation. Mol. Cell.

[45] Janna A., Davarinejad H., Joshi M., Couture J.F. (2020). Structural paradigms in the recognition of the nucleosome core particle by histone lysine methyltransferases. Front. Cell Dev. Biol..

[46] Worden E.J., Wolberger C. (2019). Activation and regulation of H2B-Ubiquitin-dependent histone methyltransferases. Curr. Opin. Struct. Biol..

[47] Wood A., Schneider J., Shilatifard A. (2005). Cross-talking histones: Implications for the regulation of gene expression and DNA repair. Biochem. Cell Biol..

[48] Smith E., Shilatifard A. (2010). The chromatin signaling pathway: Diverse mechanisms of recruitment of histone-modifying enzymes and varied biological outcomes. Mol. Cell.

[49] Ng H.H., Xu R.M., Zhang Y., Struhl K. (2002). Ubiquitination of histone H2B by Rad6 is required for efficient Dot1-mediated methylation of histone H3 lysine 79. J. Biol. Chem..

[50] Valencia-Sánchez M.I., De Ioannes P., Wang M., Vasilyev N., Chen R., Nudler E., Armache J.P., Armache K.J. (2019). Structural basis of Dot1L stimulation by histone H2B lysine 120 ubiquitination. Mol. Cell.

[51] McGinty R.K., Kim J., Chatterjee C., Roeder R.G., Muir T.W. (2008). Chemically ubiquitylated histone H2B stimulates hDot1L-mediated intranucleosomal methylation. Nature.

[52] Min J., Feng Q., Li Z., Zhang Y., Xu R.M. (2003). Structure of the catalytic domain of human DOT1L, a non-SET domain nucleosomal histone methyltransferase. Cell.

[53] Steger D.J., Lefterova M.I., Ying L., Stonestrom A.J., Schupp M., Zhuo D., Vakoc A.L., Kim J.E., Chen J., Lazar M.A. (2008). DOT1L/KMT4 recruitment and H3K79 methylation are ubiquitously coupled with gene transcription in mammalian cells. Mol. Cell. Biol..

[54] Worden E.J., Hoffmann N.A., Hicks C.W., Wolberger C. (2019). Mechanism of Cross-talk between H2B Ubiquitination and H3 Methylation by Dot1L. Cell.

[55] Guppy B.J., McManus K.J. (2015). Mitotic accumulation of dimethylated lysine 79 of histone H3 is important for maintaining genome integrity during mitosis in human cells. Genetics.

[56] Worden E.J., Zhang X., Wolberger C. (2020). Structural basis for COMPASS recognition of an H2B-ubiquitinated nucleosome. eLife.

[57] Shilatifard A. (2012). The COMPASS family of histone H3K4 methylases: Mechanisms of regulation in development and disease pathogenesis. Annu. Rev. Biochem..

[58] Basnet H., Su X.B., Tan Y., Meisenhelder J., Merkurjev D., Ohgi K.A., Hunter T., Pillus L., Rosenfeld M.G. (2014). Tyrosine phosphorylation of histone H2A by CK2 regulates transcriptional elongation. Nature.

[59] Batta K., Zhang Z., Yen K., Goffman D.B., Pugh B.F. (2011). Genome-wide function of H2B ubiquitylation in promoter and genic regions. Genes Dev..

[60] Shema E., Kim J., Roeder R.G., Oren M. (2011). RNF20 inhibits TFIIS-facilitated transcriptional elongation to suppress pro-oncogenic gene expression. Mol. Cell.

[61] Xie W., Nagarajan S., Baumgart S.J., Kosinsky R.L., Najafova Z., Kari V., Hennion M., Indenbirken D., Bonn S., Grundhoff A. (2017). RNF40 regulates gene expression in an epigenetic context-dependent manner. Genome Biol..

[62] Hooda J., Novak M., Salomon M.P., Matsuba C., Ramos R.I., MacDuffie E., Song M., Hirsch M.S., Lester J., Parkash V. (2019). Early loss of histone H2B monoubiquitylation alters chromatin accessibility and activates key immune pathways that facilitate progression of ovarian cancer. Cancer Res..

[63] Nakamura K., Kato A., Kobayashi J., Yanagihara H., Sakamoto S., Oliveira D.V., Shimada M., Tauchi H., Suzuki H., Tashiro S. (2011). Regulation of homologous recombination by RNF20-dependent H2B ubiquitination. Mol. Cell.

[64] So C.C., Ramachandran S., Martin A. (2019). E3 Ubiquitin ligases RNF20 and RNF40 are required for double-stranded break (DSB) repair: Evidence for monoubiquitination of histone H2B lysine 120 as a novel axis of DSB signaling and repair. Mol. Cell. Biol..

[65] Shiloh Y., Shema E., Moyal L., Oren M. (2011). RNF20-RNF40: A ubiquitin-driven link between gene expression and the DNA damage response. FEBS Lett..

[66] Oliveira D.V., Kato A., Nakamura K., Ikura T., Okada M., Kobayashi J., Yanagihara H., Saito Y., Tauchi H., Komatsu K. (2014). Histone chaperone FACT regulates homologous recombination by chromatin remodeling through interaction with RNF20. J. Cell Sci..

[67] Kari V., Shchebet A., Neumann H., Johnsen S.A. (2011). The H2B ubiquitin ligase RNF40 cooperates with SUPT16H to induce dynamic changes in chromatin structure during DNA double-strand break repair. Cell Cycle.

[68] Ting X., Xia L., Yang J., He L., Si W., Shang Y., Sun L. (2019). USP11 acts as a histone deubiquitinase functioning in chromatin reorganization during DNA repair. Nucleic Acids Res..

[69] Ramachandran S., Haddad D., Li C., Le M.X., Ling A.K., So C.C., Nepal R.M., Gommerman J.L., Yu K., Ketela T. (2016). The SAGA deubiquitination module promotes DNA repair and class switch recombination through ATM and DNAPK-mediated γH2AX formation. Cell Rep..

[70] Li C., Irrazabal T., So C.C., Berru M., Du L., Lam E., Ling A.K., Gommerman J.L., Pan-Hammarström Q., Martin A. (2018). The H2B deubiquitinase Usp22 promotes antibody class switch recombination by facilitating non-homologous end joining. Nat. Commun..

[71] Mosbech A., Lukas C., Bekker-Jensen S., Mailand N. (2013). The deubiquitylating enzyme USP44 counteracts the DNA double-strand break response mediated by the RNF8 and RNF168 ubiquitin ligases. J. Biol. Chem..

[72] Kari V., Raul S.K., Henck J.M., Kitz J., Kramer F., Kosinsky R.L., Übelmesser N., Mansour W.Y., Eggert J., Spitzner M. (2019). The histone methyltransferase DOT1L is required for proper DNA damage response, DNA repair, and modulates chemotherapy responsiveness. Clin. Epigenet..

[73] Schneider D., Chua R.L., Molitor N., Hamdan F.H., Rettenmeier E.M., Prokakis E., Mishra V.K., Kari V., Wegwitz F., Johnsen S.A. (2019). The E3 ubiquitin ligase RNF40 suppresses apoptosis in colorectal cancer cells. Clin. Epigenet..

[74] Wang E., Kawaoka S., Yu M., Shi J., Ni T., Yang W., Zhu J., Roeder R.G., Vakoc C.R. (2013). Histone H2B ubiquitin ligase RNF20 is required for MLL-rearranged leukemia. Proc. Natl. Acad. Sci. USA.

[75] Atanassov B.S., Mohan R.D., Lan X., Kuang X., Lu Y., Lin K., McIvor E., Li W., Zhang Y., Florens L. (2016). ATXN7L3 and ENY2 coordinate activity of multiple H2B deubiquitinases important for cellular proliferation and tumor growth. Mol. Cell.

[76] DeVine T., Sears R.C., Dai M.S. (2018). The ubiquitin-specific protease USP36 is a conserved histone H2B deubiquitinase. Biochem. Biophys. Res. Commun..

[77] Lapinska K., Faria G., McGonagle S., Macumber K.M., Heerboth S., Sarkar S. (2018). Cancer progenitor cells: The result of an epigenetic event?. Anticancer Res..

[78] Tee W.W., Reinberg D. (2014). Chromatin features and the epigenetic regulation of pluripotency states in ESCs. Development.

[79] Vincent A., Ouelkdite-Oumouchal A., Souidi M., Leclerc J., Neve B., Van Seuningen I. (2019). Colon cancer stemness as a reversible epigenetic state: Implications for anticancer therapies. World J. Stem Cells.

[80] Wang X. (2019). Stem cells in tissues, organoids, and cancers. Cell. Mol. Life Sci..

[81] Onder T.T., Kara N., Cherry A., Sinha A.U., Zhu N., Bernt K.M., Cahan P., Marcarci B.O., Unternaehrer J., Gupta P.B. (2012). Chromatin-modifying enzymes as modulators of reprogramming. Nature.

[82] Fuchs G., Shema E., Vesterman R., Kotler E., Wolchinsky Z., Wilder S., Golomb L., Pribluda A., Zhang F., Haj-Yahya M. (2012). RNF20 and USP44 regulate stem cell differentiation by modulating H2B monoubiquitylation. Mol. Cell.

[83] Karpiuk O., Najafova Z., Kramer F., Hennion M., Galonska C., König A., Snaidero N., Vogel T., Shchebet A., Begus-Nahrmann Y. (2012). The histone H2B monoubiquitination regulatory pathway is required for differentiation of multipotent stem cells. Mol. Cell.

[84] Yan Z., Wang Z., Sharova L., Sharov A.A., Ling C., Piao Y., Aiba K., Matoba R., Wang W., Ko M.S. (2008). BAF250B-associated SWI/SNF chromatin-remodeling complex is required to maintain undifferentiated mouse embryonic stem cells. Stem Cells.

[85] Sze C.C., Cao K., Collings C.K., Marshall S.A., Rendleman E.J., Ozark P.A., Chen F.X., Morgan M.A., Wang L., Shilatifard A. (2017). Histone H3K4 methylation-dependent and -independent functions of Set1A/COMPASS in embryonic stem cell self-renewal and differentiation. Genes Dev..

[86] Wang X., Wang H., Xu B., Jiang D., Huang S., Yu H., Wu Z., Wu Q. (2019). Depletion of H3K79 methyltransferase Dot1L promotes cell invasion and cancer stem-like cell property in ovarian cancer. Am. J. Transl. Res..

[87] Breindel J.L., Skibinski A., Sedic M., Wronski-Campos A., Zhou W., Keller P.J., Mills J., Bradner J., Onder T., Kuperwasser C. (2017). Epigenetic reprogramming of lineage-committed human mammary epithelial cells requires DNMT3A and loss of DOT1L. Stem Cell Rep..

[88] Bourguignon L.Y., Wong G., Shiina M. (2016). Up-regulation of histone methyltransferase, DOT1L, by matrix hyaluronan promotes microRNA-10 expression leading to tumor cell invasion and chemoresistance in cancer stem cells from head and neck squamous cell carcinoma. J. Biol. Chem..

[89] Sussman R.T., Stanek T.J., Esteso P., Gearhart J.D., Knudsen K.E., McMahon S.B. (2013). The epigenetic modifier ubiquitin-specific protease 22 (USP22) regulates embryonic stem cell differentiation via transcriptional repression of sex-determining region Y-box 2 (SOX2). J. Biol. Chem..

[90] Yun X., Zhang K., Wang J., Pangeni R.P., Yang L., Bonner M., Wu J., Wang J., Nardi I.K., Gao M. (2018). Targeting USP22 suppresses tumorigenicity and enhances cisplatin sensitivity through ALDH1A3 downregulation in cancer-initiating cells from lung adenocarcinoma. Mol. Cancer Res..

[91] Jiang S., Song C., Gu X., Wang M., Miao D., Lv J., Liu Y. (2018). Ubiquitin-specific peptidase 22 contributes to colorectal cancer stemness and chemoresistance via Wnt/β-catenin pathway. Cell. Physiol. Biochem..

[92] Glinsky G.V., Berezovska O., Glinskii A.B. (2005). Microarray analysis identifies a death-from-cancer signature predicting therapy failure in patients with multiple types of cancer. J. Clin. Investig..

[93] Liu T., Sun B., Zhao X., Li Y., Zhao X., Liu Y., Yao Z., Gu Q., Dong X., Shao B. (2015). USP44+ cancer stem cell subclones contribute to breast cancer aggressiveness by promoting vasculogenic mimicry. Mol. Cancer Ther..

[94] Gu X., Jiang D., Wang Y., Bachmair A., He Y. (2009). Repression of the floral transition via histone H2B monoubiquitination. Plant J..

[95] Du Y., He W., Deng C., Chen X., Gou L., Zhu F., Guo W., Zhang J., Wang T. (2016). Flowering-related RING protein 1 (FRRP1) regulates flowering time and yield potential by affecting histone H2B monoubiquitination in rice (*Oryza Sativa*). PLoS ONE.

[96] Li X., Jiang Y., Ji Z., Liu Y., Zhang Q. (2015). BRHIS1 suppresses rice innate immunity through binding to monoubiquitinated H2A and H2B variants. EMBO Rep..

[97] Urasaki Y., Heath L., Xu C.W. (2012). Coupling of glucose deprivation with impaired histone H2B monoubiquitination in tumors. PLoS ONE.

[98] Shema E., Tirosh I., Aylon Y., Huang J., Ye C., Moskovits N., Raver-Shapira N., Minsky N., Pirngruber J., Tarcic G. (2008). The histone H2B-specific ubiquitin ligase RNF20/hBRE1 acts as a putative tumor suppressor through selective regulation of gene expression. Genes Dev..

[99] Dickson K.A., Cole A.J., Gill A.J., Clarkson A., Gard G.B., Chou A., Kennedy C.J., Henderson B.R., Fereday S., Traficante N. (2016). The RING finger domain E3 ubiquitin ligases BRCA1 and the RNF20/RNF40 complex in global loss of the chromatin mark histone H2B monoubiquitination (H2Bub1) in cell line models and primary high-grade serous ovarian cancer. Hum. Mol. Genet..

[100] Melling N., Grimm N., Simon R., Stahl P., Bokemeyer C., Terracciano L., Sauter G., Izbicki J.R., Marx A.H. (2016). Loss of H2Bub1 Expression is Linked to Poor Prognosis in Nodal Negative Colorectal Cancers. Pathol. Oncol. Res..

[101] Zhang K., Wang J., Tong T.R., Wu X., Nelson R., Yuan Y.C., Reno T., Liu Z., Yun X., Kim J.Y. (2017). Loss of H2B monoubiquitination is associated with poor-differentiation and enhanced malignancy of lung adenocarcinoma. Int. J. Cancer.

[102] Wang Z.J., Yang J.L., Wang Y.P., Lou J.Y., Chen J., Liu C., Guo L.D. (2013). Decreased histone H2B monoubiquitination in malignant gastric carcinoma. World J. Gastroenterol..

[103] Lee J.H., Jeon Y.G., Lee K.H., Lee H.W., Park J., Jang H., Kang M., Lee H.S., Cho H.J., Nam D.H. (2017). RNF20 suppresses tumorigenesis by inhibiting the SREBP1c-PTTG1 axis in kidney cancer. Mol. Cell. Biol..

[104] Zheng X., Chen K., Liu X., Pan Y., Liu H. (2018). High RNF40 expression indicates poor prognosis of hepatocellular carcinoma. Int. J. Clin. Exp. Pathol..

[105] Medzhitov R. (2010). Inflammation 2010: New adventures of an old flame. Cell.

[106] Abdalla L.F., Chaudhry Ehsanullah R., Karim F., Oyewande A.A., Khan S. (2020). Role of using nonsteroidal anti-inflammatory drugs in chemoprevention of colon cancer in patients with inflammatory bowel disease. Cureus.

[107] Kimmel J., Axelrad J. (2020). The complex interplay between inflammatory bowel disease and malignancy. Curr. Gastroenterol. Rep..

[108] Hirano T., Hirayama D., Wagatsuma K., Yamakawa T., Yokoyama Y., Nakase H. (2020). Immunological mechanisms in inflammation-associated colon carcinogenesis. Int. J. Mol. Sci..

[109] Greuter T., Vavricka S., König A.O., Beaugerie L., Scharl M. (2020). Malignancies in inflammatory bowel disease. Digestion.

[110] Ben-Neriah Y., Karin M. (2011). Inflammation meets cancer, with NF-κB as the matchmaker. Nat. Immunol..

[111] Hartman Z.C., Poage G.M., den Hollander P., Tsimelzon A., Hill J., Panupinthu N., Zhang Y., Mazumdar A., Hilsenbeck S.G., Mills G.B. (2013). Growth of triple-negative breast cancer cells relies upon coordinate autocrine expression of the proinflammatory cytokines IL-6 and IL-8. Cancer Res..

[112] Wegwitz F., Prokakis E., Pejkovska A., Kosinsky R.L., Glatzel M., Pantel K., Wikman H., Johnsen S.A. (2020). The histone H2B ubiquitin ligase RNF40 is required for HER2-driven mammary tumorigenesis. Cell Death Dis..

[113] Labidi-Galy S.I., Papp E., Hallberg D., Niknafs N., Adleff V., Noe M., Bhattacharya R., Novak M., Jones S., Phallen J. (2017). High grade serous ovarian carcinomas originate in the fallopian tube. Nat. Commun..

[114] Zhang S., Dolgalev I., Zhang T., Ran H., Levine D.A., Neel B.G. (2019). Both fallopian tube and ovarian surface epithelium are cells-of-origin for high-grade serous ovarian carcinoma. Nat. Commun..

[115] Chernikova S.B., Razorenova O.V., Higgins J.P., Sishc B.J., Nicolau M., Dorth J.A., Chernikova D.A., Kwok S., Brooks J.D., Bailey S.M. (2012). Deficiency in mammalian histone H2B ubiquitin ligase Bre1 (Rnf20/Rnf40) leads to replication stress and chromosomal instability. Cancer Res..

[116] Liu Z., Oh S.M., Okada M., Liu X., Cheng D., Peng J., Brat D.J., Sun S.Y., Zhou W., Gu W. (2009). Human BRE1 is an E3 ubiquitin ligase for Ebp1 tumor suppressor. Mol. Biol. Cell.

[117] Portney B.A., Khatri R., Meltzer W.A., Mariano J.M., Zalzman M. (2018). ZSCAN4 is negatively regulated by the ubiquitin-proteasome system and the E3 ubiquitin ligase RNF20. Biochem. Biophys. Res. Commun..

[118] Duan Y., Huo D., Gao J., Wu H., Ye Z., Liu Z., Zhang K., Shan L., Zhou X., Wang Y. (2016). Ubiquitin ligase RNF20/40 facilitates spindle assembly and promotes breast carcinogenesis through stabilizing motor protein Eg5. Nat. Commun..

[119] Chin L.S., Vavalle J.P., Li L. (2002). Staring, a novel E3 ubiquitin-protein ligase that targets syntaxin 1 for degradation. J. Biol. Chem..

[120] Nakachi I., Rice J.L., Coldren C.D., Edwards M.G., Stearman R.S., Glidewell S.C., Varella-Garcia M., Franklin W.A., Keith R.L., Lewis M.T. (2014). Application of SNP microarrays to the genome-wide analysis of chromosomal instability in premalignant airway lesions. Cancer Prev. Res..

[121] Barber T.D., McManus K., Yuen K.W., Reis M., Parmigiani G., Shen D., Barrett I., Nouhi Y., Spencer F., Markowitz S. (2008). Chromatid cohesion defects may underlie chromosome instability in human colorectal cancers. Proc. Natl. Acad. Sci. USA.

[122] Tahara T., Yamamoto E., Madireddi P., Suzuki H., Maruyama R., Chung W., Garriga J., Jelinek J., Yamano H.O., Sugai T. (2014). Colorectal carcinomas with CpG island methylator phenotype 1 frequently contain mutations in chromatin regulators. Gastroenterology.

[123] Varambally S., Yu J., Laxman B., Rhodes D.R., Mehra R., Tomlins S.A., Shah R.B., Chandran U., Monzon F.A., Becich M.J. (2005). Integrative genomic and proteomic analysis of prostate cancer reveals signatures of metastatic progression. Cancer Cell.

[124] Kosinsky R.L., Chua R.L., Qui M., Saul D., Mehlich D., Ströbel P., Schildhaus H.U., Wegwitz F., Faubion W.A., Johnsen S.A. (2019). Loss of RNF40 Decreases NF-κB Activity in Colorectal Cancer Cells and Reduces Colitis Burden in Mice. J. Crohn’s Colitis.

[125] Blank M., Tang Y., Yamashita M., Burkett S.S., Cheng S.Y., Zhang Y.E. (2012). A tumor suppressor function of Smurf2 associated with controlling chromatin landscape and genome stability through RNF20. Nat. Med..

[126] Fu L., Cui C.P., Zhang X., Zhang L. (2019). The functions and regulation of Smurfs in cancers. Semin. Cancer Biol..

[127] Chernikova S.B., Brown J.M. (2012). R-loops and genomic instability in Bre1 (RNF20/40)-deficient cells. Cell Cycle.

[128] Prives C., Lowe S.W. (2015). Cancer: Mutant p53 and chromatin regulation. Nature.

[129] Laptenko O., Prives C. (2017). p53: Master of life, death, and the epigenome. Genes Dev..

[130] Wu C., Cui Y., Liu X., Zhang F., Lu L.Y., Yu X. (2020). The RNF20/40 complex regulates p53-dependent gene transcription and mRNA splicing. J. Mol. Cell Biol..

[131] Argentini M., Barboule N., Wasylyk B. (2001). The contribution of the acidic domain of MDM2 to p53 and MDM2 stability. Oncogene.

[132] Minsky N., Oren M. (2004). The RING domain of Mdm2 mediates histone ubiquitylation and transcriptional repression. Mol. Cell.

[133] Zhu B., Zheng Y., Pham A.D., Mandal S.S., Erdjument-Bromage H., Tempst P., Reinberg D. (2005). Monoubiquitination of human histone H2B: The factors involved and their roles in HOX gene regulation. Mol. Cell.

[134] Mosimann C., Hausmann G., Basler K. (2006). Parafibromin/Hyrax activates Wnt/Wg target gene transcription by direct association with beta-catenin/Armadillo. Cell.

[135] Wade P.A., Werel W., Fentzke R.C., Thompson N.E., Leykam J.F., Burgess R.R., Jaehning J.A., Burton Z.F. (1996). A novel collection of accessory factors associated with yeast RNA polymerase II. Protein Expr. Purif..

[136] Carpten J.D., Robbins C.M., Villablanca A., Forsberg L., Presciuttini S., Bailey-Wilson J., Simonds W.F., Gillanders E.M., Kennedy A.M., Chen J.D. (2002). HRPT2, encoding parafibromin, is mutated in hyperparathyroidism-jaw tumor syndrome. Nat. Genet..

[137] Marsh D.J., Hahn M.A., Howell V.M., Gill A.J. (2007). Molecular diagnosis of primary hyperparathyroidism in familial cancer syndromes. Expert Opin. Med. Diagn..

[138] Howell V.M., Haven C.J., Kahnoski K., Khoo S.K., Petillo D., Chen J., Fleuren G.J., Robinson B.G., Delbridge L.W., Philips J. (2003). HRPT2 mutations are associated with malignancy in sporadic parathyroid tumours. J. Med. Genet..

[139] Shattuck T.M., Välimäki S., Obara T., Gaz R.D., Clark O.H., Shoback D., Wierman M.E., Tojo K., Robbins C.M., Carpten J.D. (2003). Somatic and germ-line mutations of the HRPT2 gene in sporadic parathyroid carcinoma. N. Engl. J. Med..

[140] Gill A.J., Clarkson A., Gimm O., Keil J., Dralle H., Howell V.M., Marsh D.J. (2006). Loss of nuclear expression of parafibromin distinguishes parathyroid carcinomas and hyperparathyroidism-jaw tumor (HPT-JT) syndrome-related adenomas from sporadic parathyroid adenomas and hyperplasias. Am. J. Surg. Pathol..

[141] Howell V.M., Gill A., Clarkson A., Nelson A.E., Dunne R., Delbridge L.W., Robinson B.G., Teh B.T., Gimm O., Marsh D.J. (2009). Accuracy of combined protein gene product 9.5 and parafibromin markers for immunohistochemical diagnosis of parathyroid carcinoma. J. Clin. Endocrinol. Metab..

[142] Gill A.J., Lim G., Cheung V.K.Y., Andrici J., Perry-Keene J.L., Paik J., Sioson L., Clarkson A., Sheen A., Luxford C. (2019). Parafibromin-deficient (HPT-JT Type, CDC73 Mutated) parathyroid tumors demonstrate distinctive morphologic features. Am. J. Surg. Pathol..

[143] Pyo J.S., Cho W.J. (2019). Diagnostic and prognostic implications of parafibromin immunohistochemistry in parathyroid carcinoma. Biosci. Rep..

[144] Juhlin C.C., Nilsson I.L., Lagerstedt-Robinson K., Stenman A., Bränström R., Tham E., Höög A. (2019). Parafibromin immunostainings of parathyroid tumors in clinical routine: A near-decade experience from a tertiary center. Mod. Pathol..

[145] Bell D., Berchuck A., Birrer M., Chien J., Cramer D., Dao F., Dhir R., DiSaia P., Gabra H., Glenn P. (2011). Integrated genomic analyses of ovarian carcinoma. Nature.

[146] Alsop K., Fereday S., Meldrum C., deFazio A., Emmanuel C., George J., Dobrovic A., Birrer M.J., Webb P.M., Stewart C. (2012). BRCA mutation frequency and patterns of treatment response in BRCA mutation-positive women with ovarian cancer: A report from the Australian Ovarian Cancer Study Group. J. Clin. Oncol..

[147] Moschetta M., George A., Kaye S.B., Banerjee S. (2016). BRCA somatic mutations and epigenetic BRCA modifications in serous ovarian cancer. Ann. Oncol..

[148] Koboldt D.C., Fulton R.S., McLellan M.D., Schmidt H., Kalicki-Veizer J., McMichael J.F., Fulton L.L., Dooling D.J., Ding L., Mardis E.R. (2012). Comprehensive molecular portraits of human breast tumours. Nature.

[149] Shiovitz S., Korde L.A. (2015). Genetics of breast cancer: A topic in evolution. Ann. Oncol..

[150] Yoshida R. (2020). Hereditary breast and ovarian cancer (HBOC): Review of its molecular characteristics, screening, treatment, and prognosis. Breast Cancer.

[151] Alenezi W.M., Fierheller C.T., Recio N., Tonin P.N. (2020). Literature Review of BARD1 as a Cancer Predisposing Gene with a Focus on Breast and Ovarian Cancers. Genes.

[152] Mallery D.L., Vandenberg C.J., Hiom K. (2002). Activation of the E3 ligase function of the BRCA1/BARD1 complex by polyubiquitin chains. EMBO J..

[153] Masliah-Planchon J., Bièche I., Guinebretière J.M., Bourdeaut F., Delattre O. (2015). SWI/SNF chromatin remodeling and human malignancies. Ann. Rev. Pathol..

[154] Alfert A., Moreno N., Kerl K. (2019). The BAF complex in development and disease. Epigenet. Chromatin.

[155] Centore R.C., Sandoval G.J., Soares L.M.M., Kadoch C., Chan H.M. (2020). Mammalian SWI/SNF Chromatin Remodeling Complexes: Emerging Mechanisms and Therapeutic Strategies. Trends Genet..

[156] Kadoch C., Hargreaves D.C., Hodges C., Elias L., Ho L., Ranish J., Crabtree G.R. (2013). Proteomic and bioinformatic analysis of mammalian SWI/SNF complexes identifies extensive roles in human malignancy. Nat. Genet..

[157] Li X.S., Trojer P., Matsumura T., Treisman J.E., Tanese N. (2010). Mammalian SWI/SNF--a subunit BAF250/ARID1 is an E3 ubiquitin ligase that targets histone H2B. Mol. Cell. Biol..

[158] Nicassio F., Corrado N., Vissers J.H., Areces L.B., Bergink S., Marteijn J.A., Geverts B., Houtsmuller A.B., Vermeulen W., Di Fiore P.P. (2007). Human USP3 is a chromatin modifier required for S phase progression and genome stability. Curr. Biol..

[159] van der Knaap J.A., Kumar B.R., Moshkin Y.M., Langenberg K., Krijgsveld J., Heck A.J., Karch F., Verrijzer C.P. (2005). GMP synthetase stimulates histone H2B deubiquitylation by the epigenetic silencer USP7. Mol. Cell.

[160] Joo H.Y., Jones A., Yang C., Zhai L., Smith A.D.t., Zhang Z., Chandrasekharan M.B., Sun Z.W., Renfrow M.B., Wang Y. (2011). Regulation of histone H2A and H2B deubiquitination and Xenopus development by USP12 and USP46. J. Biol. Chem..

[161] Long L., Thelen J.P., Furgason M., Haj-Yahya M., Brik A., Cheng D., Peng J., Yao T. (2014). The U4/U6 recycling factor SART3 has histone chaperone activity and associates with USP15 to regulate H2B deubiquitination. J. Biol. Chem..

[162] Zhang X.Y., Varthi M., Sykes S.M., Phillips C., Warzecha C., Zhu W., Wyce A., Thorne A.W., Berger S.L., McMahon S.B. (2008). The putative cancer stem cell marker USP22 is a subunit of the human SAGA complex required for activated transcription and cell-cycle progression. Mol. Cell.

[163] Lan X., Atanassov B.S., Li W., Zhang Y., Florens L., Mohan R.D., Galardy P.J., Washburn M.P., Workman J.L., Dent S.Y.R. (2016). USP44 Is an Integral Component of N-CoR that Contributes to Gene Repression by Deubiquitinating Histone H2B. Cell Rep..

[164] Zhang Z., Jones A., Joo H.Y., Zhou D., Cao Y., Chen S., Erdjument-Bromage H., Renfrow M., He H., Tempst P. (2013). USP49 deubiquitinates histone H2B and regulates cotranscriptional pre-mRNA splicing. Genes Dev..

[165] Jeusset L.M., McManus K.J. (2017). Ubiquitin Specific Peptidase 22 regulates histone H2B mono-ubiquitination and exhibits both oncogenic and tumor suppressor roles in cancer. Cancers.

[166] Schrecengost R.S., Dean J.L., Goodwin J.F., Schiewer M.J., Urban M.W., Stanek T.J., Sussman R.T., Hicks J.L., Birbe R.C., Draganova-Tacheva R.A. (2014). USP22 regulates oncogenic signaling pathways to drive lethal cancer progression. Cancer Res..

[167] Kim D., Hong A., Park H.I., Shin W.H., Yoo L., Jeon S.J., Chung K.C. (2017). Deubiquitinating enzyme USP22 positively regulates c-Myc stability and tumorigenic activity in mammalian and breast cancer cells. J. Cell. Physiol..

[168] Atanassov B.S., Dent S.Y. (2011). USP22 regulates cell proliferation by deubiquitinating the transcriptional regulator FBP1. EMBO Rep..

[169] Baker S.P., Grant P.A. (2007). The SAGA continues: Expanding the cellular role of a transcriptional co-activator complex. Oncogene.

[170] Morgan M.T., Haj-Yahya M., Ringel A.E., Bandi P., Brik A., Wolberger C. (2016). Structural basis for histone H2B deubiquitination by the SAGA DUB module. Science.

[171] Lang G., Bonnet J., Umlauf D., Karmodiya K., Koffler J., Stierle M., Devys D., Tora L. (2011). The tightly controlled deubiquitination activity of the human SAGA complex differentially modifies distinct gene regulatory elements. Mol. Cell. Biol..

[172] Bonnet J., Wang C.Y., Baptista T., Vincent S.D., Hsiao W.C., Stierle M., Kao C.F., Tora L., Devys D. (2014). The SAGA coactivator complex acts on the whole transcribed genome and is required for RNA polymerase II transcription. Genes Dev..

[173] Zhang K., Yang L., Wang J., Sun T., Guo Y., Nelson R., Tong T.R., Pangeni R., Salgia R., Raz D.J. (2019). Ubiquitin-specific protease 22 is critical to in vivo angiogenesis, growth and metastasis of non-small cell lung cancer. Cell Commun. Signal..

[174] Wang Y., Sun Q., Mu N., Sun X., Wang Y., Fan S., Su L., Liu X. (2020). The deubiquitinase USP22 regulates PD-L1 degradation in human cancer cells. Cell Commun. Signal..

[175] Cortez J.T., Montauti E., Shifrut E., Gatchalian J., Zhang Y., Shaked O., Xu Y., Roth T.L., Simeonov D.R., Zhang Y. (2020). CRISPR screen in regulatory T cells reveals modulators of Foxp3. Nature.

[176] Yang J., Wei P., Barbi J., Huang Q., Yang E., Bai Y., Nie J., Gao Y., Tao J., Lu Y. (2020). The deubiquitinase USP44 promotes Treg function during inflammation by preventing FOXP3 degradation. EMBO Rep..

[177] Zhang H.Y., Liao B.W., Xu Z.S., Ran Y., Wang D.P., Yang Y., Luo W.W., Wang Y.Y. (2020). USP44 positively regulates innate immune response to DNA viruses through deubiquitinating MITA. PLoS Pathog..

[178] Nicholson B., Suresh Kumar K.G. (2011). The multifaceted roles of USP7: New therapeutic opportunities. Cell Biochem. Biophys..

